# Child inhibited temperament and caregiver distraction encouragement jointly predict children’s delay of gratification competencies

**DOI:** 10.1038/s41598-024-52288-w

**Published:** 2024-01-20

**Authors:** Anna Luerssen, Ozge Ugurlu, Iris Mauss, Özlem Ayduk

**Affiliations:** 1 Lehman College, City University of New York, 113 Gillet Hall, 250 Bedford Park Blvd West, Bronx, New York USA; 2 University of California, Berkeley, Berkeley, California USA

**Keywords:** Human behaviour, Psychology

## Abstract

A cool attentional focus during the classic delay of gratification (DG) task involves shifting attention away from the emotion-arousing features and is a key mechanism that underlies children’s ability to resist temptation and wait. Yet, we know relatively little about what gives rise to individual differences in cool focus in the first place. The current study (*N* = 162, *M*_age_ = 6.86 years) addressed this question by focusing on key aspects of child temperament (i.e., behavioral inhibition, BI) and caregiver emotion socialization (i.e., distraction encouragement) as joint predictors of cool focus. We theorized that because children are left alone in an unfamiliar environment for an undefined duration, the DG task would be especially taxing for children higher in BI, hindering their ability to deploy a cool focus and wait. We also reasoned that caregiver encouragement of distraction would serve as a protective factor by allowing children higher in BI to more easily activate a cool focus even when experiencing a taxing task. Results were partially consistent with these hypotheses, shedding new light on precursors to a central ingredient of DG ability.

## Introduction

Delay of gratification (DG) is the ability to put off a less desirable but immediately available reward in order to receive a more desirable reward later^1^, and has been linked to myriad social-cognitive competencies in early childhood and throughout development^2^. For example, those who waited longer for two treats (e.g., marshmallows, cookies) rather than having one right away during the classic laboratory paradigm of DG (heretofore, the DG task) in early childhood, went on to have higher SAT scores, better socioemotional skills, fewer behavioral problems, and were more likely to pursue advanced educational degrees in later life^2–6^. Although these longitudinal associations may be more robust in some populations than others^5,7^, the large body of research on DG suggests that it is a meaningful individual difference with important implications for achievement and well-being.

Research examining the mechanisms underlying successful waiting during the DG task point to the relevance of attentional control as children manage the emotionally-arousing nature of the experience^8–12^. In this context, effective attentional control involves shifting attention away from the emotionally evocative, tempting, or “hot” features of the situation and instead focusing on more abstract, neutral, or “cool” features^8,13^. A “cool” attentional focus may involve reappraisal which can be achieved by attending to the rewards’ informational (e.g., shape, color; “*marshmallows look like white puffy clouds*”) rather than emotion-arousing (e.g., taste; “*marshmallow are yummy and chewy*”) features. It can also be achieved by devising a cognitive or behavioral distraction during the waiting period (e.g., looking away from the rewards, thinking fun thoughts, singing a favorite song), which is the focus of the current research.

The value of a cool attentional focus has been demonstrated in experimental research with the DG task in which children are assigned to different attentional strategies and wait time is measured^8–11^. It has also been supported using an individual differences approach, in which children’s spontaneous attentional focus during the waiting period is scored alongside their wait time^12,14^. Across both of these methods, a cool attentional focus is connected to longer waiting during the DG task. Furthermore, the ability to deploy a cool focus has been shown to be a developmental precursor to inhibitory control in adulthood and linked to the functionality of neural networks that support effortful self-control^15,16^.

### Predictors of delay of gratification competencies

Given the ostensible importance of DG competencies across development, including the role of a cool attentional focus in effective waiting, a central question that follows is where these individual differences come from. That is, why does one child distract themselves by counting ceiling tiles (i.e., higher cool focus) thereby enhancing their ability to wait, whereas another picks up the treat and takes a whiff of its sweet smell (i.e., lower cool focus) thereby making it harder? Spontaneous attentional focus during the DG task is connected to certain demographic and cognitive characteristics such as age and IQ, with older children and those with higher IQs tending to employ more cool-focused attention^12^. Yet, little is known about the etiology of attentional control in the context of DG beyond these factors. That this is a critical gap comes further into focus when one considers that attentional control positively contributes to cognitive, behavioral, and emotional control more generally in childhood and throughout the lifespan^17–19^.

#### Child temperament

Prior research suggests that one key source of variation in these DG competencies may be intrapersonal, including a child’s biology or temperament. Negative emotion has been shown to have a destabilizing role during the DG task^20^. As such, children with a temperament that makes them prone to negative emotion, such as those higher in behavioral inhibition, may be especially susceptible to DG difficulties. Behavioral inhibition (BI) is an aspect of temperament in early childhood characterized by emotional and physiological reactivity in the flight-or-fight system when encountering unfamiliar objects, people, or situations^21^. Here, the unfamiliar tends to be appraised as threatening^21–24^, even if there is not an overt threat in actuality, and the resultant reactivity is associated with withdrawal-related behaviors^25^.

Behavioral inhibition is relevant given its association with a variety of emotion-related processes and difficulties. This includes heightened emotional reactivity, particularly anxiety, fear, and distress, during novel encounters^25–27^. It also includes impairments in regulation when negative emotions are experienced, including underutilization of attentional control and problem-solving behaviors^28–31^. Not surprisingly, BI is linked to socioemotional vulnerabilities over time, including high negative affectivity (e.g., sadness, fear, shame) and internalizing problems (e.g., anxiety disorders)^27–35^.

Given this emotional profile, there is reason to believe BI might also be linked to difficulties with DG. On the one hand, during the DG task, highly inhibited children, like others, have to manage the desire for the immediate option and the frustration associated with waiting. Yet, given that during the task children are left alone in a strange room for an unspecified amount of time, it is likely to provoke additional emotional distress for inhibited children. Said another way, given that children higher in BI are threatened by unfamiliar situations, an added layer of temptation may be to withdraw from the situation thereby ending their distress^25^. This supposition is supported by the attachment literature, in which children characterized by a fearful temperament (similar to BI) experience higher levels of distress during the separation phase of the strange situation procedure^36^. In both the DG task and this separation phase, the child is isolated from caregivers, interacts with a stranger, and is left alone in a strange laboratory room for an unspecified amount of time. Given the overlap in the psychological features of the two tasks, we therefore reasoned that the DG task itself will activate additional distress (beyond the frustration associated with waiting) for children higher in BI, and may, therefore, be especially taxing. Indeed, this may be comparable to DG dilemmas in everyday life, such as when a highly inhibited child goes to a birthday party with unfamiliar strangers; the immediate temptation may be to leave, despite the potential fun to be had, and longer-term social benefits, if they stay. To review, given that negative emotion can make delaying gratification more challenging^20^, that children higher in BI are prone to negative emotion^25–27^, and that the DG task may be emotionally-triggering for children higher in BI^36^, there is reason to believe that these children may have a particularly hard time delaying gratification.

#### The protective role of caregiver emotion socialization

Despite the potential connection between BI and DG difficulties, there is also reason to believe that features of the caregiving environment may moderate this association. This is because caregivers play an essential role in emotion socialization, including their children’s understanding, experience, and expression of emotion, and, of interest here, their children’s emotion regulation^37^. Given the theoretical and empirical links between cool focus and DG waiting^10–12^, we hypothesized that caregiver emotion socialization in the form of distraction encouragement may be particularly beneficial to inhibited children during unfamiliar or emotionally-evocative situations such as the DG task. That is, children higher in BI might be protected against DG difficulties if their caregivers have already encouraged them to use distraction in everyday life as it allows them to deploy a cool focus during the DG task itself.

This possibility is consistent with a large literature demonstrating that caregivers’ emotion socialization is essential for the development of children's emotion regulation abilities more generally. This research suggests that one of the central challenges of infancy and early childhood is the maturation of self regulation and emotion regulation skills. Resolving this challenge involves a progression from relatively complete reliance on caregivers to the gradual advancement of more independent forms of self-control^38–41^. Caregiver influence over this process is accomplished through a variety of means including how they respond to their children’s emotion, the emotional tenor they foster in the family home, as well as the emotion regulation strategies they model and encourage^42^.

Indeed, caregivers have been shown to differ in their emotion socialization beliefs and behaviors^37^, sometimes referred to as meta-emotion philosophies^43^, and these differences have downstream consequences for children’s emotion and their regulatory control. For example, some caregivers behave as “emotional coaches,” validating and labeling their children’s emotions as well as engaging in collaborative problem-solving regarding how negative emotions can be regulated^43^. Children of these “emotion coaches” seem better able to self-soothe physiologically and show more effective emotion regulation skills years later^43^.

Even more relevant to the current discussion, research has shown that variation in caregiver emotion socialization also matters for DG waiting^20^. For example, children wait longer during DG dilemmas when raised by caregivers who react to their emotions in a responsive way, with warmth and affection, rather than by being overcontrolling or disengaged^44–46^. Though research on the mechanisms underlying this association is somewhat limited, scholars have theorized that responsive caregivers may do a better job at helping to mitigate their children’s negative emotions and modeling effective emotion regulation strategies^20^.

Overall, these developmental models and empirical findings emphasize the role of caregivers in children’s early emotion socialization as well as their self-control ability^20,38–42^. Therefore, there is good reason to believe that caregivers also play a key role in emotion socialization through the encouragement of distraction as a way to regulate their children’s negative emotions. For example, if their child is scared before a checkup at the doctor’s office, some caregivers might suggest that their child read a favorite book in the waiting room. If their child is angry that they did not get a toy they wanted, some caregivers might take out a puzzle they could play with instead. In examples such as these, caregivers attempt to externally regulate their children’s negative emotions by encouraging distraction. Presumably, over time, children may internalize this approach, independently distracting themselves during DG dilemmas and other emotionally-evocative events.

Although it is clear that caregivers play a role in emotion socialization in children with all backgrounds and temperaments, it seems reasonable to infer that this socialization may be even more beneficial to, or essential for, those children who are emotionally reactive, including those higher in BI. Indeed, research finds that caregivers have the potential to modulate the trajectory of their child’s BI over time^31,47–50^, with some of this research narrowing in on emotion socialization practices. For example, how caregivers respond to their highly inhibited children’s emotion matters for the emotion regulatory strategies their children develop as well as their social anxiety outcomes years later^30,31^. Moreover, the value of attentional control for buffering highly inhibited children against negative outcomes has been demonstrated in prior research. For example, Eisenberg and colleagues^28^ showed that though temperamentally shy children are more prone to internalizing emotions, this is only true for children who are also low in attention shifting abilities.

### The current research

Putting this all together, our model therefore suggests that although highly inhibited children may be especially vulnerable to DG difficulties given their reactive emotional profiles (particularly to the unfamiliar), caregivers may be able to buffer these difficulties by socializing them to regulate their emotions through distraction. Testing this model was the primary aim of the current research. More pointedly, in the current study, we hypothesized that children higher in BI will be more likely to use a cool attentional focus during the DG task, and to wait longer, to the degree that their caregivers have encouraged them to use distraction during everyday evocative events.

To evaluate our model, we enrolled children 5–8 years-of-age (*n* = 162) in a laboratory study. We measured children’s cool focus and waiting behavior in the standardized and validated laboratory DG task^10,12^ that allowed for an objective assessment of these constructs. We measured child BI through caregiver-reports using items from a widely-used and well-validated instrument^51^. In addition, caregivers completed a validated questionnaire^52^ assessing the degree to which they encourage the use of distraction during a series of emotionally evocative scenarios.

## Methods

### Participants

We recruited 171 children from elementary schools, museums, and local family events. Participants had to be (1) between ages 5 years (0 months) and 8 years (12 months), (2) typically developing as reported by the caregiver (i.e., children with ADHD or developmental delays were excluded), and (3) fluent in English. We chose this age range given that self-control skills have sufficiently matured by elementary school to be of relevance to the current assessment^53,54^, all participants were of elementary school-age (i.e., a constant), and criterion wait time in the DG task (more information below) is also constant across these ages. The first five were pilot participants and were not included in the analyses. There were 162 children in the final sample (*M* = 6.86 years, *SD* = 1.11). Children were accompanied to the laboratory by a caregiver (81.01% mothers, 18.35% fathers, 0.63% other). As reported by caregivers, children were 47.53% female and 52.47% male, with 49.03% white, 12.90% Asian American, 3.87% Black/African American, 0.65% Native Hawaiian or Pacific Islander, 27.10% multiracial, and 6.45% identifying in another way. Of the children in this sample, 16.34% were Hispanic/Latinx. Regarding caregiver educational background, 6.41% had a high school diploma or GED, 7.69% had an associate’s degree, 41.03% had a bachelor’s degree, 28.21% had a master’s degree, 10.26% had a doctorate, and 6.41% had a professional degree (MD, JD, etc.)

### Procedures

#### Overview

The current research is an integral part of a larger parent study whose main aim was to examine links between self-control, emotion reasoning/regulation, personality, and parenting. Questionnaire items relevant to the current assessment are outlined in the supplemental materials as are a list of all additional tasks and questionnaires in the parent study. At the outset of the laboratory session, the caregiver completed informed consent and parental permission and the child completed assent. Subsequently, the experimenter accompanied the child to a separate room where they completed the DG task. The child then completed a series of additional tasks and questionnaires. The caregiver completed a background questionnaire which included the measures below. The session took 1 to 1.5 h. Children were compensated with a small toy of their choice.

#### Delay of gratification task

Following standard procedures^10^, the child was asked if they preferred to have one cookie or two. After establishing preference for the larger reward, the experimenter placed the two options on a plate in front of the child and explained the rules. If the child could wait without leaving their seat or touching/tasting the cookies while the experimenter was out of the room, then the child could have the two cookies. If the child did not want to wait any longer, they could bring the experimenter back by ringing a bell. However, if they rang the bell, they could only have one cookie. After making sure the child understood the contingencies, the experimenter left the room which signaled the onset of the waiting period. The waiting period ended if the child broke any of the contingencies, rang the bell, or after the full 15-min waiting criterion. Participants who preferred a single cookie (*n* = 21) were excluded from all DG analyses. Note that they were not different from the rest of the sample on baseline variables (child inhibition, caregiver distraction encouragement) or demographic characteristics (age, gender) (*ts* < 1.57*, ps* > 0.12). Participants who were familiar with the delay task (*n* = 2) were also excluded (additional exclusions outlined below).

### Measures

#### Delay of gratification task

Three coders agreed on the onset and offset of the waiting period for each participant and calculated wait time. In cases in which a procedural error impacted a participant’s wait time measurement, the participant was excluded from the analyses (*n* = 6). Of the final wait time sample (*n* = 133), 47.37% waited the full 15-min period.

Coders trained on established attentional coding procedures^12,14^, in which participants’ attention is scored second-by-second for whether they are looking at the reward, bell, or elsewhere (or missing when attention was obscured). When it was challenging to label eye gaze, coders made their best guess and rated their confidence on a scale from 1 (*not sure at all*) to 3 (*almost certain*). Seconds in which confidence was scored as 1 were recoded as missing. Coders trained on four randomly selected participants (IRR = 0.72–0.93) and then scored the full sample. Final codes were based on the agreement of at least two of the three coders (in rare situations in which all three disagreed, codes were replaced as missing) (total missing = 0.04%).

Cool focus was scored as seconds attending elsewhere divided by the total codable seconds. Once again, if a procedural error impacted attentional coding, the participant was excluded from the cool focus analysis (*n* = 5). In addition, following prior work^55^, participants whose wait time was less than 5 s were removed from the analysis given that with such little codable data the proportion calculated is a relatively unreliable measurement (*n* = 8). There were 126 participants in the final cool focus analyses. Neither age nor gender (1 = male, − 1 = female) were related to exclusion from wait time (age: *r* = 0.06, *p* = 0.450; gender: *r* = − 0.07, *p* = 0.366) or cool focus (age: *r* = − 0.01, *p* = 0.906; gender: *r* = − 0.06, *p* = 0.478) analyses.

#### Caregiver questionnaire

The caregiver questionnaire included the following measures which are relevant to the current assessment. Due to concerns about questionnaire length, many of the validated measures were shortened to include a smaller set of subscales or items. In addition, to facilitate feasibility, we utilized a 5-item rating scale for all of the questions.

##### Child behavioral inhibition

Child BI was assessed with a modified version of The Child Behavior Questionnaire—Very Short-Form^51^, a well-validated and widely-used caregiver-reported measure of child temperament. As explained above, we only included 15 of the original items which were measured on a scale from 1 (*strongly disagree*) to 5 (*strongly agree*). Three of these items were chosen on a theoretical basis because they best mapped onto the conceptual definition of BI: “my child takes a long time in approaching new situations,” “my child is sometimes shy, even around people s/he has known for a long time,” and “my child seems to be at ease with almost anyone” (reverse-scored). To evaluate the factor structure of the 15 chosen items, we conducted an exploratory factor analysis with varimax rotation. As predicted, the three items intended to capture BI loaded on the same factor (loadings ranged from 0.70 to 0.83). No other items loaded on the BI factor above 0.30 and the three items did not load on any other factor above 0.30. As such, we averaged scores on these items to create a composite measure of BI (α = 0.73). Due to time constraints, nine caregivers did not report on their child’s BI.

##### Caregiver distraction encouragement

Caregiver distraction encouragement was measured with a modified version of The Coping with Children’s Negative Emotions Scale^52^ which assesses caregivers’ responses to their children’s negative emotions. The original scale includes 12 emotion-laden scenarios but was shortened to six scenarios. For each scenario, the caregiver is presented with six coping responses and asked to indicate how they would respond to each on a scale from 1 (*very unlikely*) to 5 (*very likely*). Of relevance, the “emotion-focused reactions” subscale measures the extent to which the respondent distracts the child from the emotion-eliciting trigger (heretofore referred to as “distraction encouragement”). As an example, caregivers were asked: “If my child is about to appear in a recital or sports activity and becomes visibly nervous about people watching him/her, I would,” and distraction encouragement was assessed with the item: “Suggest that my child think about something relaxing so that his/her nervousness will go away.” We selected scenarios based on two considerations. First, we wanted to represent a range of common emotional situations and therefore included scenarios assessing sadness, anxiety, and fear. Second, for some scenarios, the emotion-focused reaction item assesses caregiver comforting rather than distraction encouragement and these scenarios were not included. To create a single measure, we averaged scores across the six scenarios (α = 0.67). Again, due to time constraints, six caregivers did not report on their distraction encouragement. See the supplemental materials for more information on this measure.

### Ethical approval

All study procedures were approved by the University of California, Berkeley Institutional Review Board. All methods were carried out in accordance with relevant guidelines and regulations.

## Results

### Preliminary analyses

Table 1 presents descriptive statistics and zero-order correlations for all study variables. As expected, there was a positive correlation between cool focus and wait time, such that the more a child distracted themselves from the rewards and bell, the longer they were able to wait.

**Table 1:** Descriptive statistics and zero-order correlations.

	Variable	M (SD)	Range	1	2	3	4	5	6
1	Cool focus	0.79 (0.14)	0.33–1.0	–	0.60**	− 0.07	0.08	0.41**	− 0.20*
2	Wait time (s)	608.95 (352.92)	0–899	–	–	0.05	0.04	0.24**	− 0.10
3	Behavioral inhibition	2.70 (0.96)	1.0–5.0	–	–	–	0.04	− 0.04	− 0.12
4	Distraction encouragement	3.89 (0.68)	1.83–5.0	–	–	–		− 0.05	− 0.08
5	Age (years)	6.86 (1.11)	5.0–8.92	–	–	–	–	–	0.02
6	Gender	–	–	–	–	–	–	–	–

Gender 1 = male, − 1 = female; * *p* < 0.05, ** *p* < 0.01. Cool focus and wait time pertain to the delay of gratification task. Behavioral inhibition was measured with items from The Child Behavior Questionnaire—Very Short Form^51^. Distraction encouragement was measured with a modified version of The Coping with Children’s Negative Emotions Scale^52^.

### Data analytic plan

For our primary analyses, we evaluated the effect of child BI and caregiver distraction encouragement as well as their interaction in predicting DG cool focus and wait time. For cool focus, we conducted general linear model analysis using the GLM procedure in SAS. Given that there was a ceiling effect in our wait time variable (47.37% of participants waited the criterion time), we elected to use tobit analysis (also called a censored regression model), which is appropriate when there is censoring in the dependent variable. This was accomplished with the QLIM procedure in SAS. Predictors were mean-centered and significant interactions were followed up with simple slopes analyses that examined a) the effect of caregiver distraction encouragement among children lower vs. higher in BI (1 *SD* below/above the mean), and b) the effect of BI among children whose caregivers were lower vs. higher (1 *SD* below/above the mean) in distraction encouragement.

Consistent with the broader DG literature^12,56,57^, age was positively correlated with both cool focus and wait time and was included as a covariate across analyses. Some research has shown a female advantage in DG wait time^58^, but we did not find that here. Rather, girls were higher in cool focus than boys (*M*_*girls*_ = 0.82, *SD* = 0.13; *M*_*boys*_ = 0.76, *SD* = 0.15), which is inconsistent with past research^14,15^. As such, we present results for cool focus with and without gender as a covariate. Please note that there are minor changes in the reported degrees of freedom across the analyses due to missing values.

### Primary analyses

**Figure 1:**
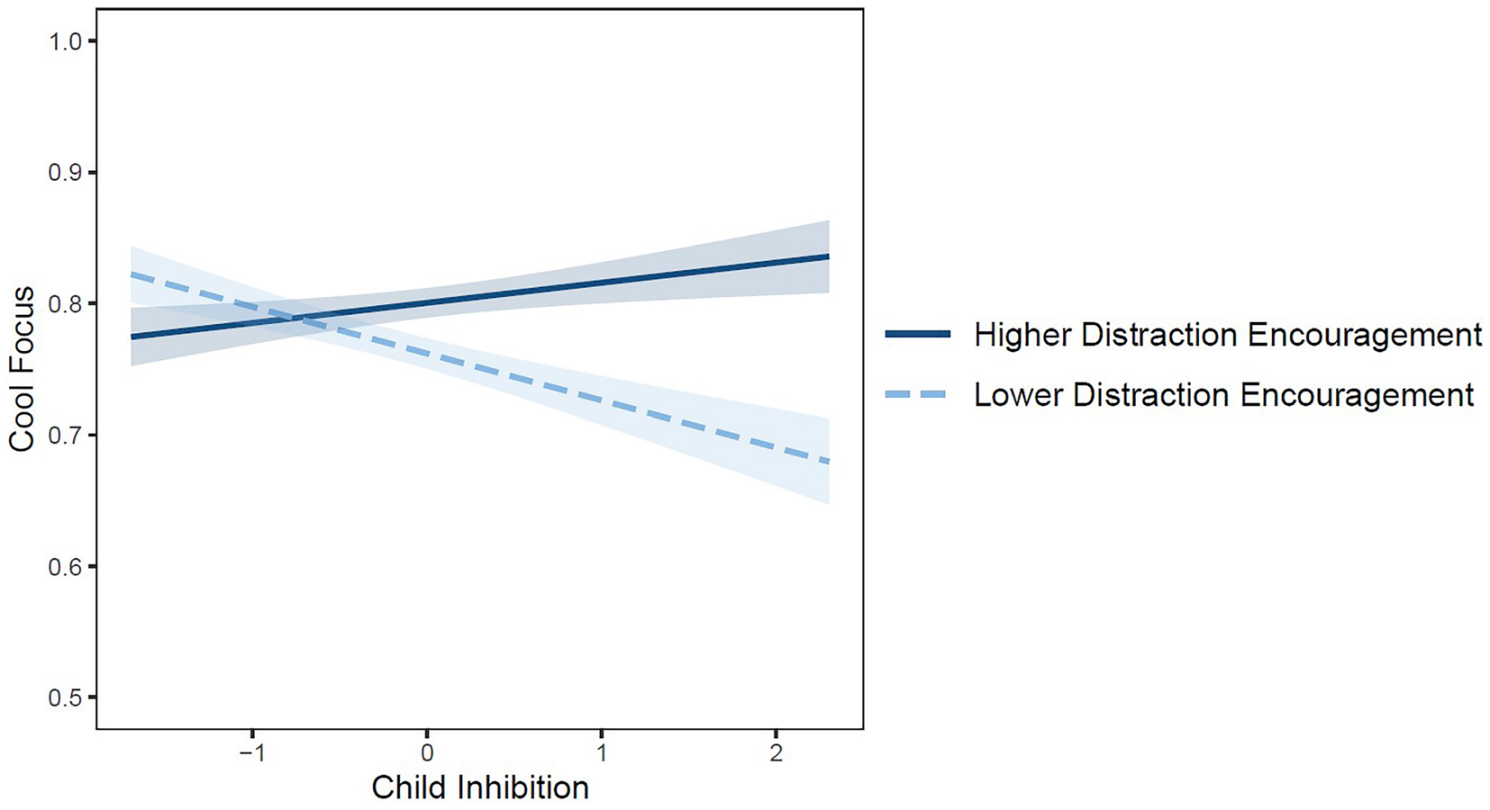
Cool focus pertains to the delay of gratification task. Child behavioral inhibition was measured with items from The Child Behavior Questionnaire—Very Short Form^51^. Caregiver distraction encouragement was measured with a modified version of The Coping with Children’s Negative Emotions Scale^52^. Both behavioral inhibition and distraction encouragement were mean-centered. Predicted values are one standard deviation above and below the mean of caregiver distraction encouragement. Highly inhibited children were significantly higher in cool focus during the DG task to the degree that their caregivers encouraged distraction during everyday emotionally evocative events.

For cool focus, there was no effect of BI, *F*(1, 115) = 0.56, *p* = 0.455, b = − 0.009, 95% CI [− 0.03, 0.01], $\eta_{p}^{2}$ = 0.005, no effect of caregiver distraction encouragement, *F*(1, 115) = 2.58, *p* = 0.111, b = 0.03, 95% CI [− 0.006, 0.06], $\eta_{p}^{2}$ = 0.02, but a significant BI by distraction encouragement interaction, *F*(1, 115) = 4.34, *p* = 0.040, b = 0.04, 95% CI [0.002, 0.07], $\eta_{p}^{2}$ = 0.04, which significantly improved the model fit over a main-effects only model (ΔR^2^ = 0.03, *p* = 0.040). As can be seen in Fig. 1, simple slopes analyses showed that for less inhibited children, there was no difference in cool focus as a function of distraction encouragement, *F*(1, 115) = 0.11, *p* = 0.740, b = − 0.007, 95% CI [− 0.05, 0.04], $\eta_{p}^{2}$ = 0.001. In contrast, highly inhibited children whose caregivers encouraged distraction were significantly more cool focused during the waiting period, *F*(1, 115) = 5.82, *p* = 0.018, b = 0.06 , 95% CI [0.01, 0.11], $\eta_{p}^{2}$ = 0.05. Moreover, BI was unrelated to cool focus in children whose caregivers were higher in distraction encouragement, *F*(1, 115) = 0.88, *p* = 0.349, b = 0.02 , 95% CI [− 0.02, . = 051], $\eta_{p}^{2}$ = 0.01. Although also nonsignificant, descriptively, BI appeared negatively associated with cool focus in children whose caregivers were lower in distraction encouragement, *F*(1, 115) = 3.75, *p* = 0.055, b = − 0.03 , 95% CI [− 0.07, 0.0007], $\eta_{p}^{2}$ = 0.03. In separate analyses, there were no two- or three-way interactions with the primary predictors and child age in predicting cool focus.

When gender was added as an additional covariate the interaction became nonsignificant, *F*(1, 114) = 2.87, *p* = 0.093, b = 0.03, 95% CI [− 0.005, 0.06], $\eta_{p}^{2}$ = 0.02, although the pattern was in the same direction. Again, in separate analyses, there were no two- or three-way interactions with the primary predictors and child gender in predicting cool focus.

For wait time, tobit analysis showed that there was no significant effect of child BI, *t* = 0.97, *p* = 0.331, b = 54.34, caregiver distraction encouragement, *t* = 1.07, *p* = 0.29, b = 80.74, or a BI X distraction encouragement interaction, *t* = 1.41, *p* = 0.158, b = 105.38. In separate analyses, there were no significant two- or three-way interactions with child age.

## Discussion

Performance on the classic DG task in early childhood is connected to a host of social-cognitive competencies across development^2–6^, and cool attentional focus during the waiting period is a significant antecedent of whether and how long a child will wait^8–12^. As such, in the current research, we explored predictors of cool focus (as well as DG wait time), particularly the interactive effects of child temperament (i.e., BI) and caregiver emotion socialization (i.e., distraction encouragement). Our model suggested that higher BI in children, which is connected to emotional reactivity during unfamiliar situations^25–27^, may be associated with added challenge during the DG task as it involves being alone in an unfamiliar environment. Yet, caregivers may buffer such difficulty to the degree that they encourage their children to use distraction to regulate emotions in everyday life.

The results of this study provide some evidence consistent with our model in that child BI and caregiver distraction encouragement interacted in predicting cool focus. In unpacking the meaning of this interaction, we found that distraction encouragement was unrelated to cool focus in less inhibited children. This fits with our theorizing that lower levels of BI may be associated with less dependence on caregivers to actively participate in emotion regulation, at least in children who are 5–8 years old. Interestingly, there was not a direct association between BI and distraction encouragement which suggests that even though caregivers of children lower (vs. higher) in BI were just as likely to encourage distraction, children lower in BI may be less dependent on that socialization to engage in distraction during the DG task themselves. By contrast, highly inhibited children utilized a cool attentional focus more during the DG task to the degree that their caregiver encouraged distraction. Caregiver distraction encouragement seems to have better prepared them for the challenges inherent in the DG task, in particular by promoting spontaneous shifting of attention to cool features of the environment rather than the “hot” rewards and bell, where the latter not only represent an enticement but also a way to withdraw from the stressful situation.

In this sample, girls were higher in cool focus than boys (though gender was unrelated to wait time), an effect inconsistent with the broader literature on attentional deployment in the context of DG^14,15^. Nevertheless, we note that although the pattern of results for cool focus was largely unchanged when gender was included as an additional covariate, the interaction did become nonsignificant. While this may reflect a reduction in power from adding another predictor to the model, future research is needed to better elucidate the nuanced relationship between gender and these variables. It is also important to reiterate that there was no interaction between child BI and caregiver distraction encouragement in predicting DG wait time, though descriptively the effects were in the predicted direction and paralleled the cool focus findings. This is perhaps not surprising given that the ceiling effect in our wait time data is likely to have reduced power. Indeed, studies often show significant results for attentional variables in the absence of wait time given the skewed distribution typical in samples with older children^14,15^.

That said, in an attempt to further elucidate the relationship between BI, distraction encouragement, and DG competencies, we also conducted an exploratory moderated mediation analysis that is outlined in the supplemental materials. Briefly, and consistent with our predictions, results showed that cool focus mediates the relationship between BI and DG wait time (higher BI is associated with lower cool focus, which is, in turn, associated with shorter wait times), but that this mediation only appears for children whose caregivers are lower in distraction encouragement. By contrast, higher caregiver distraction encouragement appeared to protect inhibited children against this maladaptive pathway. Though there are limitations to this analysis (e.g., measures were assessed concurrently, DG wait time was censored), and should therefore be treated as preliminary and exploratory, the results fit with prior research that has established attention deployment as a key mechanism underlying DG behavior^8–12^, and point to the value of conducting additional longitudinal or experimental research to further understand the nuanced relationship between DG competencies and these predictive factors.

Prior research demonstrates the important role caregivers play in emotion socialization in early childhood^37–43^, especially for children who are at risk of socioemotional difficulties, such as those higher in BI^30,31^. That caregiver distraction encouragement was associated with the way these children managed their attention during the DG task is consistent with this growing literature. Importantly, because caregivers’ socialization beliefs and practices are amenable to change, with downstream benefits for children^47,59^, this result points to caregiver distraction encouragement as a possible target for prevention and intervention efforts. That said, this possibility needs to be considered in light of the documented costs of using distraction indiscriminately, particularly in hindering affective habituation and cognitive restructuring, and thus, its potential role in impeding adaptive emotional processing in the longer-term^60^.

Although the results were broadly consistent with our predictions, we note several limitations of the study that need to be kept in mind. First, we focused specifically on emotion socialization in the form of distraction encouragement given its direct theoretical link to cool attentional focus in the DG task and we measured cool focus using physical eye gaze (i.e., time spent looking away from the rewards/bell). Although this measurement approach is consistent with prior research^12,14,15^, DG performance most likely involves additional, albeit less observable, strategies such as cognitive distraction or neutral reappraisal. Because it is difficult to behaviorally code for such internal mental processes, they remain unaccounted for in our research. Future research might assess these strategies via alternative methods, such as post-assessment interviews.

Second, we made several modifications to the CCNES (reducing the number of scenarios; truncating the rating scale) which might have resulted in slightly lower reliability (α = 0.67) than the typical benchmark (α = 0.70). Because lower reliability means more noise and lower statistical power, the reported effects of caregiver distraction encouragement might be underestimates of the true effect size, and therefore, should be replicated with the original scale in future studies. Additionally, we relied on caregivers’ reports of their own distraction encouragement as well as their child’s BI, a relatively common approach when studying families with young children, but one that might inflate their association due to common method variance (though note that they were uncorrelated in our sample). Nevertheless, future research could benefit from evaluating whether caregivers’ self-reports align with measurable behavior. Finally, a premise underlying the current research is that the DG task may be emotionally triggering for children higher in BI, which may then interfere with performance given the damaging effects of negative emotions^20^. While we found support for our model of cool focus, we did not measure emotional reactivity during the task directly so this premise should be evaluated with caution.

Despite these limitations, the current study represents a contribution to our understanding of DG which is connected to a variety of important outcomes across the lifespan^2^. More specifically, this study is part of a relatively small literature exploring predictors of individual differences in DG competencies, particularly cool attentional focus, a critical antecedent of DG behavior. Our results highlight that both intrapersonal and interpersonal factors may contribute to DG competencies and that a thorough understanding of DG origins must consider these contributions simultaneously. Here, we show that caregiver encouragement of distraction is one seemingly effective route to helping more reactive children, such as those higher in BI, become better able to meet the demands of DG challenges, common and impactful experiences in children’s lives.

### Supplementary Information

Supplementary Information.

## Data Availability

Study measures are outlined in the supplemental materials. Participants did not consent to posting their anonymized data on public websites, however, we are able to provide the data to interested parties on an individual basis. Please contact the lead author directly with these requests.
