# Supplementary material for: Child inhibited temperament and caregiver distraction encouragement jointly predict children’s delay of gratification competencies
Source: Sci Rep. 2024 Jan 20;14:1798. doi: 10.1038/s41598-024-52288-w (PMC10799904; doi:10.1038/s41598-024-52288-w)
Supplement: Supplementary file 1 — Supplementary Information. [file 41598_2024_52288_MOESM1_ESM.docx]

Child inhibited temperament and caregiver distraction encouragement jointly predict children’s delay of gratification competencies

**SUPPLEMENTAL MATERIALS**

**I. SURVEY MEASURES RELEVANT TO THE CURRENT STUDY**

- ***Child Behavioral Inhibition (shortened Child Behavior Questionnaire – Very Short Form)***

*Items with an asterisk were included in composite measure of Behavioral Inhibition; R = reverse-scored*

- - likes going down high slides or other adventurous activities.
  - *seems to be at ease with almost any person. (R)
  - prefers quiet activities to active games.
  - likes to go high and fast when pushed on a swing.
  - *takes a long time in approaching new situations.
  - *is sometimes shy even around people s/he has known a long time
  - is full of energy, even in the evening
  - likes rough and rowdy games.
  - is afraid of burglars or the "boogie man".
  - is quite upset by a little cut or bruise.
  - gets quite frustrated when prevented from doing something s/he wants to do.
  - tends to stay upset for ten minutes or longer when s/he is angry about something.
  - seems to feel depressed when unable to accomplish some task.
  - hardly ever complains when s/he is ill with a cold. (R)
  - is very difficult to soothe when s/he has become upset.
- ***Caregiver Distraction Encouragement (shortened Coping with Children’s Negative Emotions Scale)***

*Items with an asterisk were included in the measure of Caregiver Distraction Encouragement*

- - 1. If my child falls off his/her bike and breaks it, and then gets upset and cries, I would:
    - 1a. remain calm and not let myself get anxious
    - *1b. comfort my child and try to get him/her to forget about the accident
    - 1c. tell my child that he/she is over-reacting
    - 1d. help my child figure out how to get the bike fixed
    - 1e. tell my child it's OK to cry
    - 1f. tell my child to stop crying or he/she won't be allowed to ride his/her bike anytime soon
  - 2. If my child loses some prized possession and reacts with tears, I would:
    - 2a. get upset with him/her for being so careless and then crying about it
    - 2b. tell my child that he/she is over-reacting
    - 2c. help my child think of places he/she hasn't looked yet
    - *2d. distract my child by talking about happy things
    - 2e. tell him/her it's OK to cry when you feel unhappy
    - 2f. tell him/her that's what happens when you're not careful
  - 3. If my child is going over to spend the afternoon at a friend's house and becomes nervous and upset because I can't stay there with him/her, I would:
    - *3a. distract my child by talking about all the fun he/she will have with his/her friend
    - 3b. help my child think of things that he/she could do so that being at the friend's house without me wasn't scary (e.g., take a favorite book or toy with him/her)
    - 3c. tell my child to quit overreacting and being a child
    - 3d. tell the child that if he/she doesn't stop that he/she won't be allowed to go out anymore
    - 3e. feel upset and uncomfortable because of my child's reactions
    - 3f. encourage my child to talk about his/her nervous feelings
  - 4. If my child is about to appear in a recital or sports activity and becomes visibly nervous about people watching him/her, I would:
    - 4a. help my child think of things that he/she could do to get ready for his/her turn (e.g., to do some warm-ups and not to look at the audience)
    - *4b. suggest that my child think about something relaxing so that his/her nervousness will go away
    - 4c. remain calm and not get nervous myself
    - 4d. tell my child that he/she is being a child about it
    - 4e. tell my child that if he/she doesn't calm down, we'll have to leave and go home right away
    - 4f. encourage my child to talk about his/her nervous feelings
  - 5. If my child is panicky and can't go to sleep after watching a scary TV show, I would:
    - 5a. encourage my child to talk about what scared him/her
    - 5b. get upset with him/her for being silly
    - 5c. tell my child that he/she is over-reacting
    - 5d. help my child think of something to do so that he/she can get to sleep (e.g., take a toy to bed, leave the lights on)
    - 5e. tell him/her to go to bed or he/she won't be allowed to watch any more TV
    - *5f. do something fun with my child to help him/her forget about what scared him/her
  - 6. If my child is shy and scared around strangers and consistently becomes teary and wants to stay in his/her bedroom whenever family friends come to visit, I would:
    - 6a. help my child think of things to do that would make meeting my friends less scary (e.g., to take a favorite toy with him/her when meeting my friends)
    - 6b. tell my child that it is OK to feel nervous
    - *6c. try to make my child happy by talking about the fun things we can do with our friends
    - 6d. feel upset and uncomfortable because of my child's reactions
    - 6e. tell my child that he/she must stay in the living room and visit with our friends
    - 6f. tell my child that he/she is being a child

**II. OTHER SURVEY MEASURES AND TASKS IN PARENT STUDY**

- Demographics (date of birth, gender, race, ethnicity, native language, age since living in the U.S., zip code, marital status, working status, highest degree earned, main daily activities/responsibilities, most recent job title, number of children and adults living in household, family income, home ownership/rental status, relationship to child participating in the study, child’s date of birth, child race, child ethnicity, child’s first language)

***Caregiver Personality/Well-Being/Beliefs***

- Ten Item Personality Inventory^1^
- Single Item Self-Esteem Scale^2^
- Implicit Beliefs about Emotion^3^ – shortened
- Implicit Beliefs about 3-Year Old’s Emotion Control^3^ – (adapted, shortened)
- Brief Tangney Questionnaire^4^– plus Short Grit Scale^5^ and Emotion-Regulation Checklist^6^ items
- Past, Present, and Future Subjective SES^7^

***Caregiving Quality***

- Parent version of the Children’s Reports of Parental Behavior Inventory^8^
- Communication Patterns (self-generated face-valid items)
- Strategies for Delay of Gratification Scale – Part 1 (self-generated face-valid items)
- Strategies for Delay of Gratification Scale – Part 2 (self-generated face-valid items)
- Parenting Routines Questionnaire
- Perceived Stress Scale^9^

***Caregiver Ratings of Child Emotion/Emotion Regulation***

- The Coping with Children’s Negative Emotions Scale^10^
- Strengths and Difficulties Questionnaire^11^ – plus Emotion-Regulation Checklist^6^ items
- Child Behavior Questionnaire – Very Short-Form^12^
- Children’s Sensitivity to Punishment Sensitivity to Rewards Questionnaire^13^ – shortened

***Caregiver Ratings of Child Self-Control/Executive Function***

- Brief Tangney Questionnaire^4^ – modified for children – plus Emotion-Regulation Checklist^6^ items

***Child Self-Report Ratings***

- Pictorial Scale of Perceived Competence and Social Acceptance for Young Children^14^ – Peer Acceptance and Maternal Acceptance subscales
- Perceived Maternal Warmth and Criticism^15^
- Self-Perception Profile for Children^16^ – Global Self-Worth subscale

***Measures of Child Self-Control and Emotion Reasoning***

- Delay of gratification task^17^ (see main text)
- Emotion labeling task (developed by the authors)
- Mind-in-the-eyes task^18^
- Emotion recognition from vocal bursts task^19^
- Affective theory of mind task^20^

**III. COPING WITH CHILDREN’S NEGATIVE EMOTIONS SCALE - SUPPLEMENTAL INFORMATION AND ANALYSES**

Later research evaluating the psychometric properties of the Coping with Children’s Negative Emotions Scale (CCNES)^10^ suggested that emotion-focused reactions (i.e., distraction encouragement) and problem-focused reactions (another coping subscale) may actually load on the same factor. However, when we factor analyzed these items together, we did not find a one-factor solution. Given this, along with the conceptual distinction between these two types of emotion regulation, we elected to focus our analyses on the distraction encouragement composite as intended. That said, as can be seen in the analyses below, the pattern of results was largely the same when we combined these two subscales into a single composite (*M* = 4.18, *SD* = .51, α = .76).

For cool focus, there was a nonsignificant effect of BI**,** *F*(1, 115) = .93, *p* = .337, b = -.01, 95% CI [-.04, .01], $\eta_{p}^{2}$= .008, a nonsignificant effect of caregiver distraction/problem-focused encouragement, *F*(1, 115) = 1.02, *p* = .315, b = .02, 95% CI [-.02, .07], $\eta_{p}^{2}$= .009, and a significant BI by distraction/problem-focused encouragement interaction, *F*(1, 115) = 4.39, *p* = .038, b = .05, 95% CI [.003, .10], $\eta_{p}^{2}$= .04. Simple slopes analyses showed that for less inhibited children, there was no difference in cool focus as a function of distraction/problem-focused encouragement, *F*(1, 115) = .80, *p* = .372, b = -.03, 95% CI [-.09, .03], $\eta_{p}^{2}$= .007. In contrast, more inhibited children whose caregivers encouraged distraction and problem-focused reactions were significantly more cool-focused during the waiting period, *F*(1, 115) = 4.30, *p* = .040, b = .07 , 95% CI [.003, .14], $\eta_{p}^{2}$= .04. In separate analyses, there were no two- or three-way interactions with the primary predictors (child BI, caregiver distraction/problem-focused encouragement) and child age in predicting cool focus. When gender was added as an additional covariate, the interaction became marginal, *F*(1, 114) = 3.28, *p* = .073, b = .05, 95% CI [-.004, .10], $\eta_{p}^{2}$= .03, though the pattern was in the same direction. Again, in separate analyses, there were no two- or three-way interactions with the primary predictors and child gender in predicting cool focus.

For wait time, tobit analysis showed that there was no effect of child BI, *t* = .73, *p* = .463, b = 41.46, or caregiver distraction/problem-focused encouragement, *t* = .46, *p* = .643, b = 47.97, and no BI X caregiver-distraction/problem-focused encouragement interaction, *t* = 1.85, *p* = .064, b = 208.98. Again, in separate analyses, there were no two- or three-way interactions between child age and these primary variables in predicting wait time.

**IV. MODERATED MEDIATION**

In addition to the primary regression analyses reported in the main text, we further explored the relationship between BI, distraction encouragement, cool focus, and DG wait time by conducting an exploratory moderated mediation analysis. Based on prior research in which cool focus has been established as a causal mechanism of DG performance,^21-23^ we reasoned that cool focus should mediate the relationship between child BI and DG wait time. However, based on our theorizing, and the corresponding result that caregiver distraction encouragement protects children higher in BI against failure to deploy a cool attentional focus, we also reasoned that the mediation would be moderated by caregiver distraction encouragement. More specifically, our model suggests that BI would relate to lower cool focus, which, in turn, would predict lower DG wait times - but only among children whose caregivers were lower in distraction encouragement (i.e., those lacking in the protective factor).

To conduct the moderated mediation, we used bootstrapping procedures with the Mediation package in R. Age was included as a covariate and all variables (except the outcome, i.e., DG wait time) were mean-centered. Behavioral inhibition was specified as the predictor, cool focus as the mediator, and DG wait time as the outcome, with distraction encouragement as the moderator of the a path (BI → cool focus) and the c path (BI→ DG wait time). To aid in the interpretation of path coefficients, cool focus was converted from a proportion to a percentage for these analyses. Estimates reported in the parentheses are 95% confidence intervals.

The results revealed that the mediation model was significantly moderated by caregiver distraction encouragement, ACME: *b* = −69.53 (−138.09 ⎼ −4.15), *p* = .038, such that the mediation through cool focus was negative and marginally different from zero among children with lower (-1SD) caregiver distraction encouragement, ACME: *b* = −46.88 (−98.58 ⎼ .30), *p* = .05, but was positive and not significant among those with higher (+1 SD) caregiver distraction encouragement, ACME: *b* = 22.30 (−22.11 ⎼ 69.07, *p* = .33). See Figure S1. These results suggest that greater caregiver distraction encouragement in everyday life may help to protect children higher in BI against this maladaptive pathway.

We acknowledge that there are limitations to this exploratory analysis in that the variables were assessed concurrently and DG wait time was censored. Although the total effect of BI on DG wait time was not significant (lower distraction encouragement: *b* = −26.41 (−111.77 ⎼ 58.9, *p* = .549; higher distraction encouragement: *b* = 57.21 (−24.67 ⎼ 138.43, *p* = .16), (consistent with the main analyses reported in the manuscript), some experts suggest that significance of the individual pathways is not a precondition for mediation; what matters instead is the presence of the indirect effect.^28^

Despite these issues, these results fit with existing research showing cool focus as a key causal mechanism underlying DG waiting,^21-23^ as well as work showing that self-control competencies (operationalized as distraction encouragement) serve a protective role specifically for those most vulnerable to emotional difficulties,^24-27^ here, children higher BI.


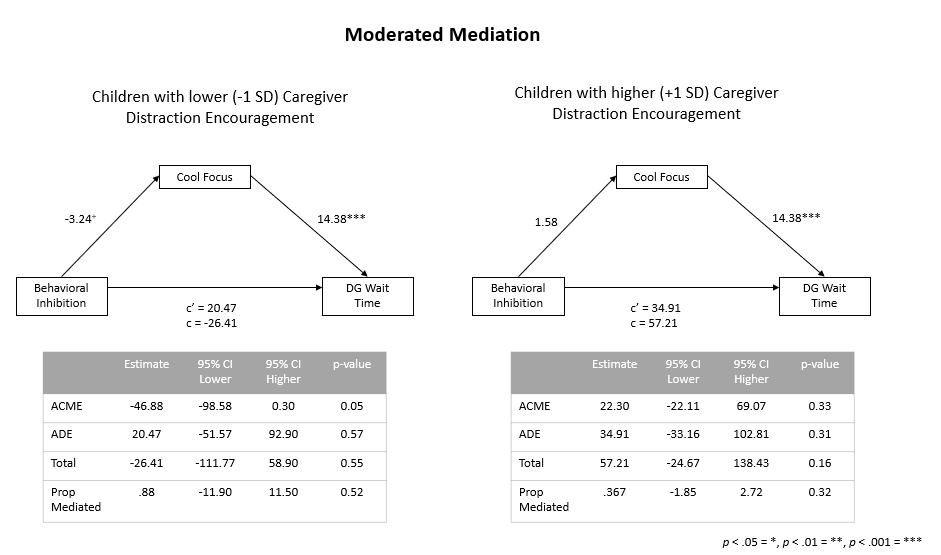


Figure S1. Cool focus and DG wait time pertain to the delay of gratification task. Child behavioral inhibition was measured with items from The Child Behavior Questionnaire – Very Short Form. Caregiver distraction encouragement was measured with a modified version of The Coping with Children’s Negative Emotions Scale.

**V. REFERENCES**

1. Gosling, S. D., Rentfrow, P. J., & Swann Jr., W. B. A very brief measure of the Big-Five personality domains. *J.Research in Personality*. **37,** 504-528 (2003).

2. Robins, R. W., Hendin, H. M., & Trzesniewski, K. H. Measuring global self-esteem: Construct validation of a single-item measure and the Rosenberg Self-Esteem Scale. *Personality and Social Psy. Bulletin*. **27,** 151-161 (2001).

3. Tamir, M., John, O. P., Srivastava, S., & Gross, J. J. Implicit theories of emotion: Affective and social outcomes across a major life transition. *J. Personality and Social Psy.* **92(4),** 731-744 (2007).

4. Tangney, J. P., Baumeister, R. F., & Boone, A. L. High self-control predicts good adjustment, less pathology, better grades, and interpersonal success. *J. Personality*. **72(2),** 271-324 (2004).

5. Duckworth, A. L., & Quinn, P. D. Development and validation of the Short Grit Scale (GRIT–S). *J. Personality Assessment*. **91,** 166-174 (2009).

6. Shields, A., & Cicchetti, D. Emotion regulation among school-age children: The development and validation of a new criterion Q-sort scale. *Developmental Psy.* **33,** 906-916 (1997).

7. Ostrove, J. M., Adler, N. E., Kuppermann, M., & Washington, A. E. Objective and subjective assessments of socioeconomic status and their relationship to self-rated health in an ethnically diverse sample of pregnant women. *Health Psy*. **19(6),** 613-618 (2000).

8. Schludermann, S. M., & Schludermann, E. H. Sociocultural change and adolescents' perceptions of parent behavior. *Developmental Psy.* **19(5),** 674-685 (1983).

9. Cohen, S., Kamarck, T., & Mermelstein, R. A global measure of perceived stress. *J. Health and Social Behavior*. **24(4),** 385-396 (1983).

10. Fabes, R., Poulin, R. E., Eisenberg, N., & Madden-Derdich, D. A. The Coping with Children’s Negative Emotions Scale (CCNES): Psychometric properties and relations with children’s emotional competence. *Marriage and Family Review*. **34(3–4),** 285-310 (2002).

11. Goodman, R. The Strengths and Difficulties Questionnaire: A research note. *J. Child Psychology and Psychiatry, and Allied Disciplines*. **38(5),** 581-586 (1997).

12. Putnam, S. P., & Rothbart, M. K. Development of short and very short forms of the Children’s Behavior Questionnaire. *J. Personality Assessment*. **87(1),** 102-112 (2006).

13. O’Connor, R. M., Colder, C. R., & Hawk Jr., L. W. Confirmatory factor analysis of the Sensitivity to Punishment and Sensitivity to Reward Questionnaire. *Personality and Individual Differences*. **37,** 985-1002 (2004).

14. Harter, S., & Pike, R. The Pictorial Scale of Perceived Competence and Social Acceptance for Young Children. *Child Development*. **55(6),** 1969-1982 (1984). https://doi.org/10.2307/1129772

15. Hooley, J. M., & Teasdale, J. D. Predictors of relapse in unipolar depressives: Expressed emotion, marital distress, and perceived criticism. *J. Abnormal Psy.* **98,** 229-235 (1989).

16. Harter, S. Manual for the Self-Perception Profile for Children. Denver: University of Denver. (1985).

17. Mischel, W., & Ebbesen, E. B. Attention in delay of gratification. *J Personality and Social Psy.* **16,** 329-337 (1970).

18. Baron-Cohen, S., Jolliffe, T., Mortimore, C., & Robertson, M. Another advanced test of theory of mind: Evidence from very high functioning adults with autism or asperger syndrome. *J. Child Psychology and Psychiatry*. **38(7),** 813-822 (1997).

19. Sauter, D. A., Panattoni, C., & Happé, F. Children’s recognition of emotions from vocal cues: Emotions in the voice. *British J. Developmental Psy.* **31(1),** 97-113 (2013).

20. Baron-Cohen, S., Leslie, A. M., & Frith, U. Does the autistic child have a “theory of mind”? *Cognition*. **21(1),** 37-46 (1985).

21. Mischel, W., & Ebbesen, E. B. Attention in delay of gratification. *J. Personality and Social Psy.* **16,** 329-337 (1970).

22. Mischel, W., Ebbesen, E., & Zeiss, A. Cognitive and attentional mechanisms in delay of gratification. *J. Personality and Social Psy.* **21(2),** 204-218 (1972).

23. Rodriguez, M. L., Mischel, W., & Shoda, Y. Cognitive person variables in the delay of gratification of older children at risk. *J. Personality and Social Psy.* **57(2),** 358-367 (1989).

24. Ayduk, Ö. *et al*. Regulating the interpersonal self: Strategic self-regulation for coping with rejection sensitivity. *J. Personality and Social Psy.* **79(5),** 776-792 (2000).

25. Ayduk, Ö. *et al*. Rejection sensitivity and executive control: Joint predictors of Borderline Personality features. *J. of Research in Personality*. **42,** 151-168 (2008).

26. Gyurak, A., & Ayduk, Ö. Defensive physiological reactions to rejection: The effect of self-esteem and attention control on startle responses. *Psy. Science*. **18,** 886-892 (2007).

27. Gyurak, A., & Ayduk, Ö. Resting respiratory sinus arrhythmia buffers against rejection sensitivity via emotion control. *Emotion*, **4,** 458-467 (2008).

28. Hayes, A. F. *Introduction to Mediation, Moderation, and Conditional Process Analysis, Second Edition: A Regression-Based Approach 3rd Edition.* (Guilford, 2022).
